# Environmental and epigenetic regulation of *Rider* retrotransposons in tomato

**DOI:** 10.1371/journal.pgen.1008370

**Published:** 2019-09-16

**Authors:** Matthias Benoit, Hajk-Georg Drost, Marco Catoni, Quentin Gouil, Sara Lopez-Gomollon, David Baulcombe, Jerzy Paszkowski

**Affiliations:** 1 The Sainsbury Laboratory, University of Cambridge, Cambridge, United Kingdom; 2 Department of Plant Sciences, University of Cambridge, Cambridge, United Kingdom; Gregor Mendel Institute of Molecular Plant Biology, AUSTRIA

## Abstract

Transposable elements in crop plants are the powerful drivers of phenotypic variation that has been selected during domestication and breeding programs. In tomato, transpositions of the LTR (long terminal repeat) retrotransposon family *Rider* have contributed to various phenotypes of agronomical interest, such as fruit shape and colour. However, the mechanisms regulating *Rider* activity are largely unknown. We have developed a bioinformatics pipeline for the functional annotation of retrotransposons containing LTRs and defined all full-length *Rider* elements in the tomato genome. Subsequently, we showed that accumulation of *Rider* transcripts and transposition intermediates in the form of extrachromosomal DNA is triggered by drought stress and relies on abscisic acid signalling. We provide evidence that residual activity of *Rider* is controlled by epigenetic mechanisms involving siRNAs and the RNA-dependent DNA methylation pathway. Finally, we demonstrate the broad distribution of *Rider-like* elements in other plant species, including crops. Our work identifies *Rider* as an environment-responsive element and a potential source of genetic and epigenetic variation in plants.

## Author summary

Transposons are major constituents of plant genomes and represent a powerful source of internal genetic and epigenetic variation. For example, domestication of maize has been facilitated by a dramatic change in plant architecture, the consequence of a transposition event. Insertion of transposons near genes often confers quantitative phenotypic variation linked to changes in transcriptional patterns, as documented for blood oranges and grapes. In tomato, the most widely grown fruit crop and model for fleshy fruit biology, occurrences of several beneficial traits related to fruit shape and plant architecture are due to the activity of the transposon family *Rider*. While *Rider* represents a unique endogenous source of genetic and epigenetic variation, mechanisms regulating *Rider* activity remain unexplored. By achieving experimentally-controlled activation of the *Rider* family, we shed light on the regulation of these transposons by drought stress, signalling by phytohormones, as well as epigenetic pathways. Furthermore, we reveal the presence of *Rider-like* elements in other economically important crops such as rapeseed, beetroot and quinoa. This suggests that drought-inducible *Rider* activation could be further harnessed to generate genetic and epigenetic variation for crop breeding, and highlights the potential of transposon-directed mutagenesis for crop improvement.

## Introduction

Transposable elements (TEs) replicate and move within host genomes. Based on their mechanisms of transposition, TEs are either DNA transposons that use a cut-and-paste mechanism or retrotransposons that transpose through an RNA intermediate via a copy-and-paste mechanism [1]. TEs make up a significant part of eukaryotic chromosomes and are a major source of genetic instability that, when active, can induce deleterious mutations. Various mechanisms have evolved that protect plant genomes, including the suppression of TE transcription by epigenetic silencing that restricts TE movement and accumulation [2–5].

Chromosomal copies of transcriptionally silenced TEs are typically hypermethylated at cytosine residues and are associated with nucleosomes containing histone H3 di-methylated at lysine 9 (H3K9me2). In addition, they are targeted by 24-nt small interfering RNAs (24-nt siRNAs) that guide RNA-dependent DNA methylation (RdDM), forming a self-reinforcing silencing loop [6–8]. Interference with these mechanisms can result in the activation of transposons. For example, loss of DNA METHYLTRANSFERASE 1 (MET1), the main methyltransferase maintaining methylation of cytosines preceding guanines (CGs), results in the activation of various TE families in Arabidopsis [9–11] and in rice [12]. Mutation of CHROMOMETHYLASE 3 (CMT3), mediating DNA methylation outside CGs, triggers the mobilization of several TE families, including *CACTA* elements in Arabidopsis [10] and *Tos17* and *Tos19* in rice [13]. Interference with the activity of the chromatin remodelling factor DECREASE IN DNA METHYLATION 1 (DDM1), as well as various components of the RdDM pathway, leads to the activation of specific subsets of TEs in Arabidopsis. These include DNA elements *CACTA* and *MULE*, as well as retrotransposons *ATGP3*, *COPIA13*, *COPIA21*, *VANDAL21*, *EVADÉ* and *DODGER* [14–17]. Similarly, loss of *OsDDM1* genes in rice results in the transcriptional activation of TE-derived sequences [18].

In addition to interference with epigenetic silencing, TE activation can also be triggered by environmental stresses. In her pioneering studies, Barbara McClintock denoted TEs as “controlling elements”, thus suggesting that they are activated by genomic stresses and are able to regulate the activities of genes [19, 20]. In the meantime, a plethora of stress-induced TEs have been described, including retrotransposons. For example, the biotic stress-responsive *Tnt1* and *Tto1* families in tobacco [21,22], the cold-responsive *Tcs* family in citrus [23], the virus-induced *Bs1* retrotransposon in maize [24], the heat-responsive retrotransposons *Go-on* in rice [25], and *ONSEN* in Arabidopsis [26,27]. While heat-stress is sufficient to trigger *ONSEN* transcription and the formation of extrachromosomal DNA (ecDNA), transposition was observed only after the loss of siRNAs, suggesting that the combination of impaired epigenetic control and environmental stress is a prerequisite for *ONSEN* transposition [28]. Studies have further shown that stress-responsive TEs can affect the expression of surrounding genes, by providing novel regulatory elements and, in some cases, conferring stress-responsiveness [28–30].

The availability of high-quality genomic sequences revealed that LTR (Long Terminal Repeat) retrotransposons make up a significant proportion of plant chromosomes, from approximately 10% in Arabidopsis, 25% in rice, 42% in soybean, and up to 75% in maize [31]. In tomato (*Solanum lycopersicum*), a model crop plant for research on fruit development, LTR retrotransposons make up about 60% of the genome [32]. Despite the abundance of retrotransposons in the tomato genome, only a limited number of studies have linked TE activities causally to phenotypic alterations. Remarkably, the most striking examples described so far involve the retrotransposon family *Rider*. For example, fruit shape variation is based on copy number variation of the *SUN* gene, which underwent *Rider*-mediated trans-duplication from chromosome 10 to chromosome 7. The new insertion of the *SUN* gene into chromosome 7 in the variety “Sun1642” results in its overexpression and consequently in the elongated tomato fruits that were subsequently selected by breeders [33,34]. The *Rider* element generated an additional *SUN* locus on chromosome 7 that encompassed more than 20 kb of the ancestral *SUN* locus present on chromosome 10 [33]. This large “hybrid” retroelement landed in the fruit-expressed gene *DEFL1*, resulting in high and fruit-specific expression of the *SUN* gene containing the retroelement [34]. The transposition event was estimated to have occurred within the last 200–500 years, suggesting that duplication of the *SUN* gene occurred after tomato domestication [35].

Jointless pedicel is a further example of a *Rider*-induced tomato phenotype that has been selected during tomato breeding. This phenotypic alteration reduces fruit dropping and thus facilitates mechanical harvesting. Several independent jointless alleles were identified around 1960 [36–38]. One of them involves a new insertion of *Rider* into the first intron of the *SEPALLATA* MADS-Box gene, *Solyc12g038510*, that provides an alternative transcription start site and results in an early nonsense mutation [39]. Also, the ancestral yellow flesh mutation in tomato is due to *Rider*-mediated disruption of the *PSY1* gene, which encodes a fruit-specific phytoene synthase involved in carotenoid biosynthesis [40,41]. Similarly, the “potato leaf” mutation is due to a *Rider* insertion in the *C* locus controlling leaf complexity [42]. *Rider* retrotransposition is also the cause of the chlorotic tomato mutant *fer*, identified in the 1960s [43]. This phenotype has been linked to *Rider*-mediated disruption of the *FER* gene encoding a bHLH-transcription factor. *Rider* landed in the first exon of the gene [44,45]. Sequence analysis of the element revealed that the causative copy of *Rider* is identical to that involved in the *SUN* gene duplication [45].

The *Rider* family belongs to the *Copia* superfamily and is ubiquitous in the tomato genome [34,45]. Based on partial tomato genome sequences, the number of *Rider* copies was estimated to be approximately 2000 [34]. Previous DNA blots indicated that *Rider* is also present in wild tomato relatives but is absent from the genomes of potato, tobacco, and coffee, suggesting that amplification of *Rider* happened after the divergence of potato and tomato approximately 6.2 mya [45,46]. The presence of *Rider* in unrelated plant species has also been suggested [47]. However, incomplete sub-optimal sampling and the low quality of genomic sequence assemblies has hindered a comprehensive survey of *Rider* elements within the plant kingdom.

Considering that the *Rider* family is a major source of phenotypic variation in tomato, it is surprising that its members and their basic activities, as well as their responsiveness and the possible triggers of environmental super-activation, which explain the evolutionary success of this family, remain largely unknown. Contrary to the majority of TEs characterized to date, previous analyses revealed that *Rider* is constitutively transcribed and produces full-length transcripts in tomato [34], but the stimulatory conditions promoting reverse transcription of *Rider* transcripts that results in accumulation as extrachromosomal DNA are unknown.

To fill these gaps, we provide here a refined annotation of full-length *Rider* elements in tomato using the most recent genome release (SL3.0). We reveal environmental conditions facilitating *Rider* activation and show that *Rider* transcription is enhanced by dehydration stress mediated by abscisic acid (ABA) signalling, which also triggers accumulation of extrachromosomal DNA. Moreover, we provide evidence that RdDM controls *Rider* activity through siRNA production and partially through DNA methylation. Finally, we have performed a comprehensive cross-species comparison of full-length *Rider* elements in 110 plant genomes, including diverse tomato relatives and major crop plants, in order to characterise species-specific *Rider* features in the plant kingdom. Together, our findings suggest that *Rider* is a drought stress-induced retrotransposon ubiquitous in diverse plant species that may have contributed to phenotypic variation through the generation of genetic and epigenetic alterations induced by historical drought periods.

## Results

### Family structure of *Rider* retrotransposons in tomato

**Fig 1:**
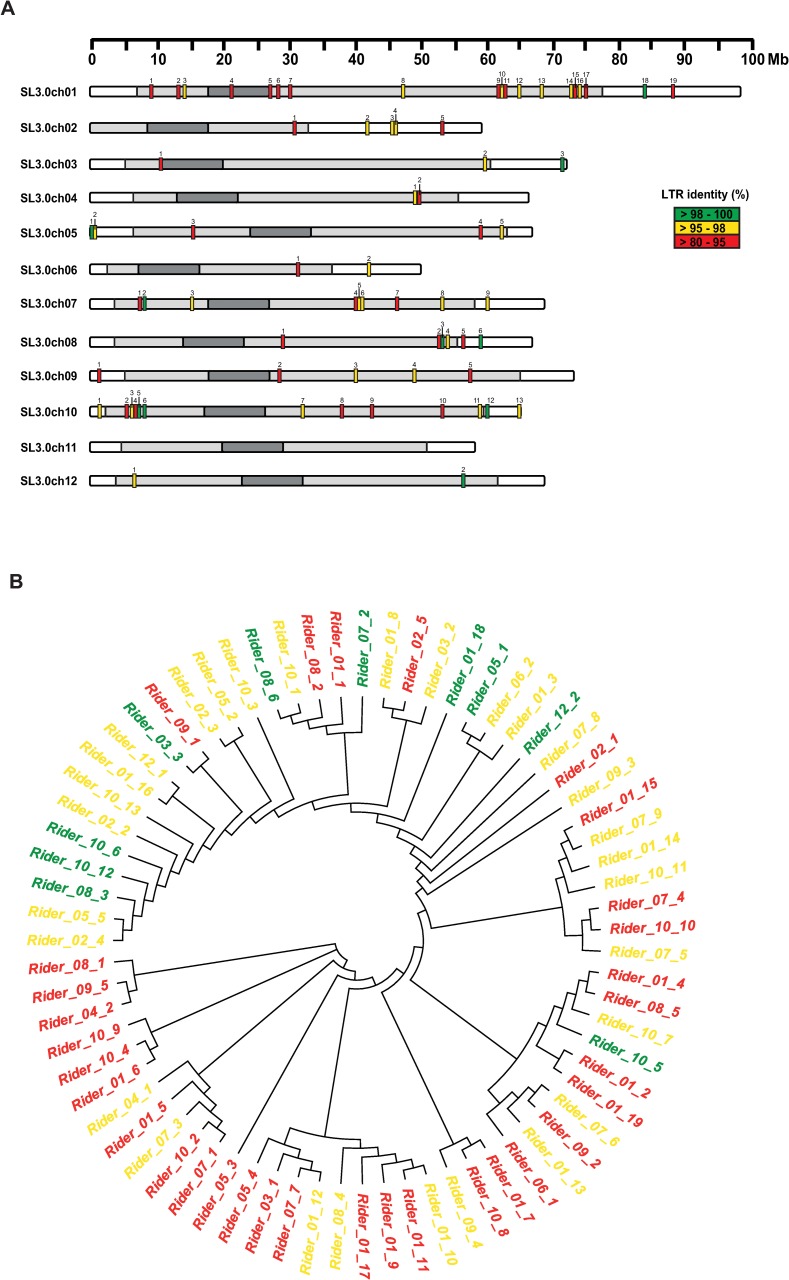
Chromosomal location and phylogenetic relationships of *de novo* annotated full-length *Rider* elements. (A) Chromosomal positions of 71 *de novo* annotated full-length *Rider* elements in the SL3.0 genome. *Rider* copies are marked as coloured vertical bars, with colours reflecting similarity between LTRs for each element. Dark grey areas delimitate the centromeres, light grey pericentromeric heterochromatin, and white euchromatin. (B) Phylogenetic relationship of the 71 *de novo* annotated *Rider* elements. The phylogenetic tree was constructed using the neighbour-joining method on nucleotide sequences of each *Rider* copy.

We used the most recent SL3.0 tomato genome release for *de novo* annotation of *Rider* elements. First, we retrieved full-length, potentially autonomous retrotransposons using our functional annotation pipeline (*LTRpred*, see Materials and Methods). We detected a set of 5844 potentially intact LTR retrotransposons (S1 Table). Homology search among these elements identified 71 elements that share >85% sequence similarity over the entire element with the reference *Rider* sequence [45] and thus belong to the *Rider* family. We then determined the distribution of these *Rider* elements along the tomato chromosomes (Fig 1A) and also estimated their age based on sequence divergence between 5’ and 3’ LTRs (Fig 1A). We classified these elements into three categories according to their LTR similarity: 80–95%, 95–98% and 98–100% (S1A Fig). While the first category contains relatively old copies (last transposition between 10.5 and 3.5 mya), the 95–98% class represents *Rider* elements that moved between 3.5 and 1.4 mya, and the 98–100% category includes the youngest *Rider* copies that transposed within the last 1.4 my (S1A Fig). Out of 71 *Rider* family members, 14 were found in euchromatic chromosome arms (14/71 or 19.7%) and 57 in heterochromatic regions (80.3%) (Table 1). In accordance with previous observations based on partial genomic sequences [34], young *Rider* elements of the 98–100% class are more likely to reside in the proximity of genes, with 50% within 2 kb of a gene. This was the case for only 37.5% of old *Rider* members (85–95% class) (Table 2). Such a distribution is consistent with the preferential presence of young elements within euchromatic chromosome arms (50%, 5/10) compared to old *Rider* elements (9.4%, 3/32) (Table 2 and S1B Fig). In addition, the phylogenetic distance between individual elements is moderately correlated to the age of each element (Fig 1B) (S2 Table).

**Table 1:** Distribution of *de novo* annotated *Rider* elements based on chromatin context.

	LTR identity (%)			
	98–100	95–98	85–95	Total (%)
Number of elements in chromosome arms	5	6	3	19.7
Number of elements in pericentromeric regions	5	23	29	80.3
Total	10	29	32	100.0

**Table 2:** Distribution of *de novo* annotated *Rider* elements based on gene proximity.

	Presence of gene within 2 kb (%)	Number of elements in chromosome arms (%)
Rider_85–95	37.5	9.4
Rider_95–98	48.3	20.7
Rider_98–100	50.0	50

### *Rider* is a drought- and ABA-responsive retrotransposon

**Fig 2:**
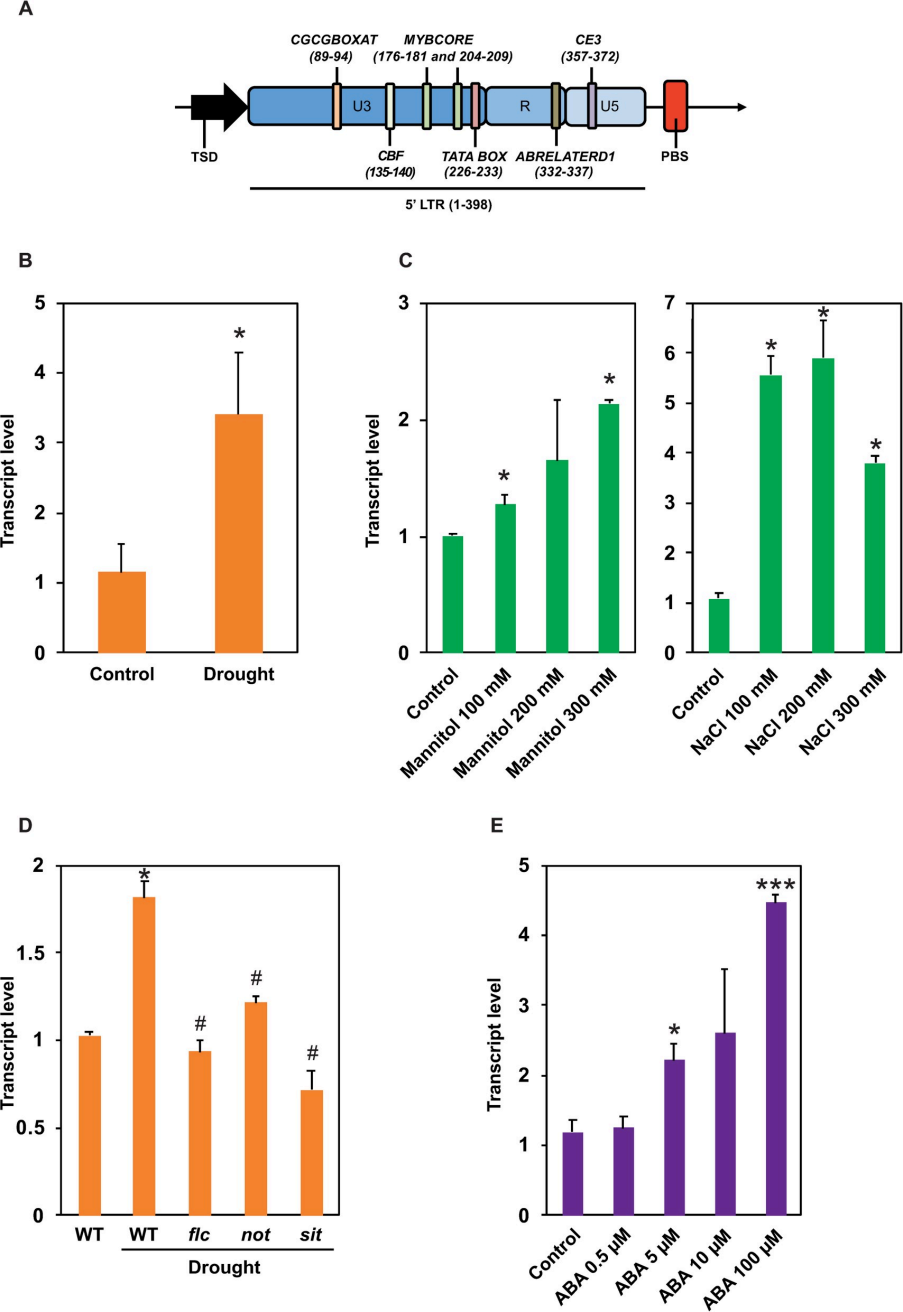
*Rider* activation is stimulated by drought and ABA. (A) Identification of *cis*-regulatory elements (CREs) within *Rider* LTRs. *Rider* LTR U3, R and U5 regions are marked, as well as neighbouring Target Site Duplication (TSD) and Primer Binding Site (PBS) sequences. CREs are marked as coloured vertical bars; their bp positions are given in brackets. (B-C) Quantification of *Rider* RNA levels by RT-qPCR in tomato seedlings after (B) drought stress or (C) mannitol and NaCl treatments. Bar charts show normalized expression relative to Control, +/- SEM from two to three biological replicates. *P<0.05, two-sided Student’s *t*-test. (D) Quantification of *Rider* RNA levels by RT-qPCR in leaves of drought-stressed tomato wild-type plants, *flc*, *not* and *sit* mutants. Bar charts show normalized expression relative to WT Control, +/- SEM from two to three biological replicates. *P<0.05 denotes difference compared to wild-type control; ^#^ *P*<0.05 denotes difference compared to wild-type drought plants, two-sided Student’s *t*-test. (E) Quantification of *Rider* RNA levels by RT-qPCR in tomato seedlings after ABA treatment. Bar charts show normalized expression relative to Control, +/- SEM from two to three biological replicates. *P<0.05, ***P<0.001, two-sided Student’s *t*-test.

To better understand the activation triggers and, thus, the mechanisms involved in the accumulation of *Rider* elements in the tomato genome, we examined possible environmental stresses and host regulatory mechanisms influencing their activity. Transcription of an LTR retroelement initiates in its 5’ LTR and is regulated by an adjacent promoter region that usually contains *cis*-regulatory elements (CREs) (reviewed in [48]). Therefore, we aligned the sequence of the *Rider* promoter region against sequences stored in the PLACE database (www.dna.affrc.go.jp/PLACE/) containing known CREs and identified several dehydration-responsive elements (DREs) and sequence motifs linked to ABA signalling (Fig 2A). First, we tested whether these CREs were present in the LTR promoter sequences of the 71 *de novo* annotated *Rider* elements (S3 Table). Comparison of *Rider* LTRs to a set of gene promoter sequences of the same length revealed significant enrichment of several CREs in *Rider* LTRs (Fisher’s exact test *P*<0.001) (S4 Table). It is known, for example, that the CGCG sequence motif at position 89–94 (Fig 2A) is recognized by transcriptional regulators binding calmodulin. These are products of signal-responsive genes activated by various environmental stresses and phytohormones such as ABA [49]. We also detected two MYB recognition sequence motifs (CTGTTG at position 176–181 bp, and CTGTTA at position 204–209 bp) (Fig 2A). MYB recognition sequences are usually enriched in the promoters of genes with transcriptional activation during water stress, elevated salinity, and ABA treatments [50,51]. In addition, an ABA-responsive element-like (ABRE-like) was found at position 332–337 bp in the R region of *Rider*’s LTR, along with a coupling element (CE3) located at position 357–372 bp (Fig 2A). The co-occurrence of ABRE-like and CE3 has often been found in ABA-responsive genes [52,53].

The simultaneous presence of these five CREs in promoters of *Rider* elements suggests that *Rider* transcription may be induced by environmental stresses such as dehydration and salinity that involves ABA mediated signalling. To test whether *Rider* transcription is stimulated by drought stress, glasshouse-grown tomato plants were subjected to water deprivation and levels of *Rider* transcripts quantified by RT-qPCR (Fig 2B). When compared to control plants, we observed a 4.4-fold increase in *Rider* transcript abundance in plants subjected to drought stress. Thus, *Rider* transcription appears to be stimulated by drought.

To further test this finding, we re-measured levels of *Rider* transcripts in different experimental setups. *In vitro* culture conditions with increasing levels of osmotic stress were used to mimic increasing drought severity (Fig 2C). Transcript levels of *Rider* increased in a dose-dependent fashion with increasing mannitol concentration, corroborating results obtained during direct drought stress in greenhouse conditions. Interestingly, tomato seedlings treated with NaCl also exhibited increased levels of *Rider* transcripts (Fig 2C).

ABA is a versatile phytohormone involved in plant development and abiotic stress responses, including drought stress [54]. Therefore, we asked whether *Rider* transcriptional drought-responsiveness is mediated by ABA and whether increased ABA can directly stimulate *Rider* transcript accumulation. To answer the first question, we exploited tomato mutants defective in ABA biosynthesis. The lines *flacca* (*flc*), *notabilis* (*not*) and *sitiens* (*sit*) have mutations in genes encoding a sulphurylase [55], a 9-cis-epoxy-carotenoid dioxygenase (*SlNCED1*) [56,57], and an aldehyde oxidase [58], respectively. Both *flc* and *sit* are impaired in the conversion of ABA-aldehyde to ABA [55,58], while *not* is unable to catalyse the cleavage of 9-cis-violaxanthin and/or 9-cis-neoxanthin to xanthoxin, an ABA precursor [57]. Glasshouse-grown *flc*, *not* and *sit* mutants and control wild-type plants were subjected to water deprivation treatment and *Rider* transcript levels quantified by RT-qPCR (Fig 2D). *Rider* transcript levels were reduced in *flc*, *not* and *sit* by 43%, 26% and 56%, respectively.

To examine whether ABA stimulates accumulation of *Rider* transcripts, tomato seedlings were transferred to media supplemented with increasing concentrations of ABA (Fig 2E). The levels of *Rider* transcripts increased in a dose-dependent manner with increasing ABA concentrations. This suggests that ABA is not only involved in signalling that results in hyper-activation of *Rider* transcription during drought, but it also directly promotes the accumulation of *Rider* transcripts. The effectiveness of the treatments was verified by assaying expression of the stress- and ABA-responsive gene *SlASR1* (S2A–S2F Fig).

Identification in the U3 region of *Rider* LTRs of a binding domain for C-repeat binding factors (CBF), which are regulators of the cold-induced transcriptional cascade [52,59], led us to test *Rider* activation by cold stress. However, *Rider* transcription was not affected by cold treatment, leaving drought and salinity as the predominant environmental stresses identified so far that stimulate accumulation of *Rider* transcripts (S2G Fig).

### RdDM regulates levels of *Rider* transcripts

**Fig 3:**
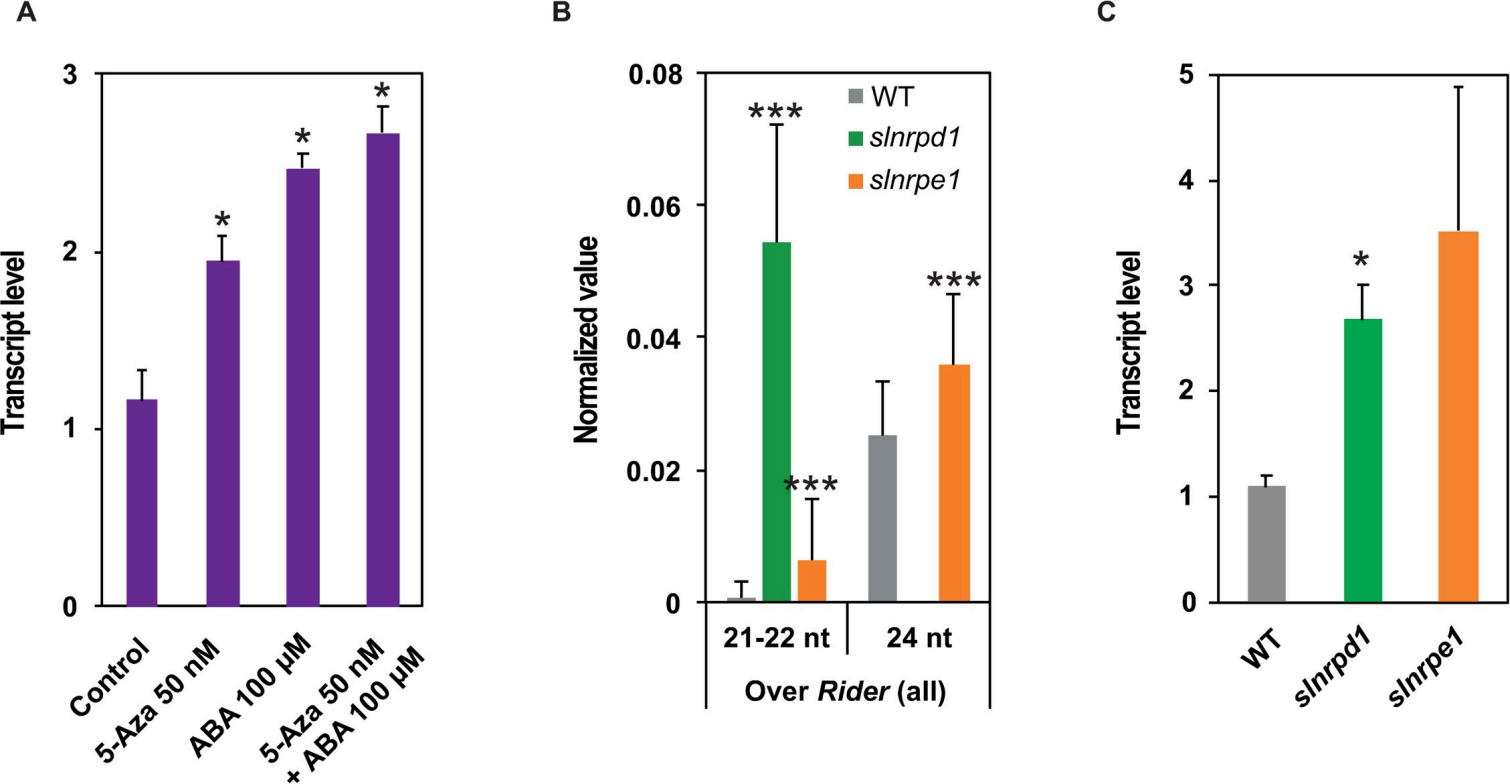
Accumulation of *Rider* transcripts in tomato plants deficient in epigenetic regulation. (A) Quantification of *Rider* RNA levels by RT-qPCR in tomato seedlings treated with 5-azacytidine and/or ABA. Bar charts show normalized expression relative to Control, +/- SEM from two to three biological replicates. *P<0.05, two-sided Student’s *t*-test. (B) Abundance of siRNAs at *Rider* elements in wild type, *slnrpd1* and *slnrpe1*. Data are expressed as siRNA reads per kb per million mapped reads and represent average normalized siRNA counts on *Rider* elements +/- SD from 71 *de novo* annotated *Rider* copies. ***P<0.001, two-sided Student’s *t*-test. (C) Quantification of *Rider* RNA by RT-qPCR in *slnrpd1* and *slnrpe1*. Bar charts show normalized expression relative to WT, +/- SEM from two to four biological replicates. *P<0.05, two-sided Student’s *t*-test.

The suppression of transposon-derived transcription by epigenetic mechanisms, which typically include DNA methylation, maintains genome integrity [2,3,5]. We asked whether *Rider* transcription is also restricted by DNA methylation. Tomato seedlings were grown on media supplemented with 5-azacytidine, an inhibitor of DNA methyltransferases. *Rider* transcript steady-state levels increased in plants treated with 5-azacytidine compared to controls (Fig 3A). Comparison of *Rider* transcript accumulation in 5-azacytidine-treated and ABA-treated plants revealed similar levels of transcripts and the levels were similar when the treatments were applied together (*P* <0.05; Fig 3A).

To further examine the role of DNA methylation in controlling *Rider* transcription, we took advantage of tomato mutants defective in crucial components of the RdDM pathway, namely SlNRPD1 and SlNRPE1, the major subunits of RNA Pol IV and Pol V, respectively. These mutants exhibit reduced cytosine methylation at CHG and CHH sites (in which H is any base other than G) residing mostly at the chromosome arms, with *slnrpd1* showing a dramatic, genome-wide loss of 24-nt siRNAs [60]. To evaluate the role of RdDM in *Rider* transcript accumulation, we first assessed the consequences of impaired RdDM on siRNA populations at full-length *Rider* elements. Deficiency in SlNRPD1 resulted in a complete loss of 24-nt siRNAs that target *Rider* elements (Fig 3B). This loss was accompanied by a dramatic increase (approximately 80-fold) in 21-22-nt siRNAs at *Rider* loci (Fig 3B). In contrast, the mutation in *SlNRPE1* triggered increases in both 21-22-nt and 24-nt siRNAs targeting *Rider* elements (Fig 3B). We then asked whether altered distribution of these siRNA classes is related to the age of the *Rider* elements and/or their chromosomal position, and thus local chromatin properties. Compilation of the genomic positions and siRNA data in RdDM mutants didn’t reveal preferential accumulation of 21-22-nt siRNAs (S3A Fig) or 24-nt siRNAs (S3B Fig) over specific *Rider* classes. Subsequently, we examined whether loss of SlNRPD1 or SlNRPE1 was sufficient to increase levels of *Rider* transcripts and observed increased accumulation of *Rider* transcripts in both *slnrpd1* and *slnrpe1* compared to WT (Fig 3C).

We assessed whether this increase in *Rider* transcript levels is linked to changes in DNA methylation levels in *Rider* elements of RdDM mutants. There was no significant change in global DNA methylation in the three sequence contexts in the 71 *de novo* annotated *Rider* elements (S3C Fig), despite a tendency for young *Rider* elements to lose CHH in *slnrpd1* and *slnrpe1* (S3D Fig). Thus, the RdDM pathway affects the levels of *Rider* transcripts. Also, features of *Rider* copies such as age and chromatin location alone cannot predict potential for activation based on DNA methylation levels.

### Extrachromosomal circular DNA of *Rider* accumulates during drought stress and in *slnrpd1* and *slnrpe1* mutants

**Fig 4:**
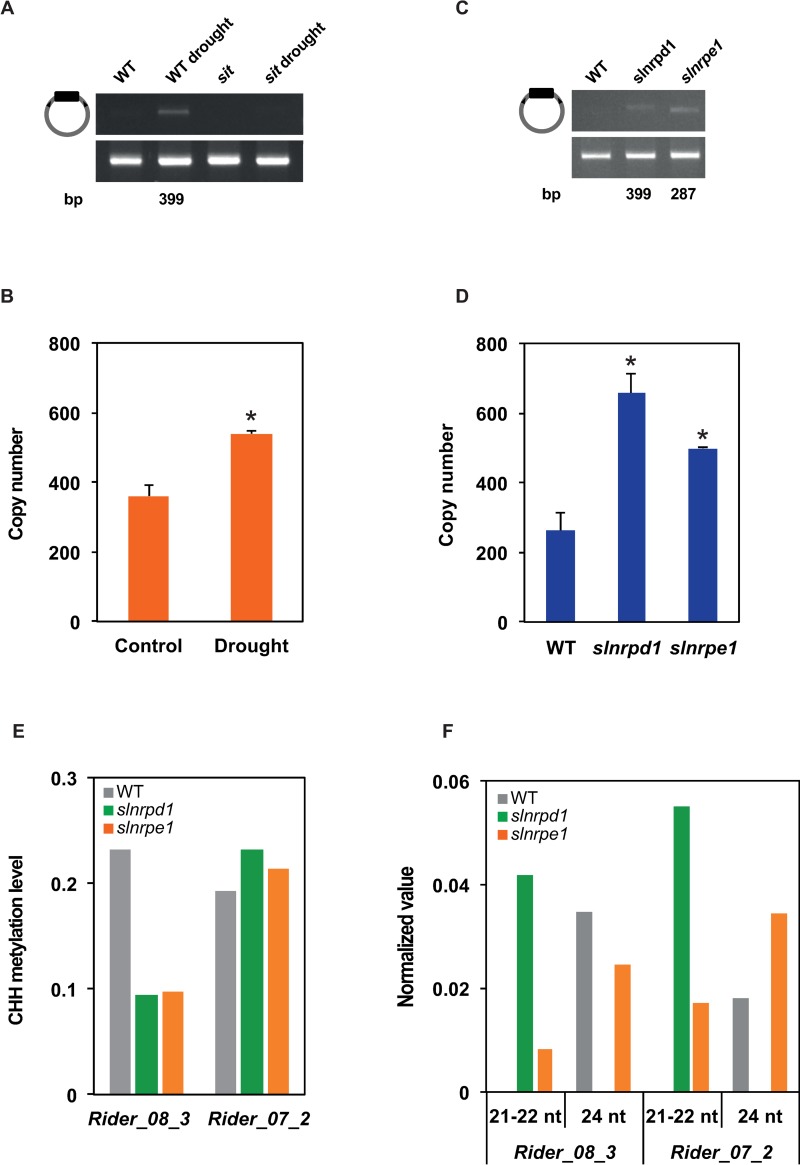
Accumulation of *Rider* extrachromosomal DNA in drought-stressed plants and in *slnrpd1* and *slnrpe1* mutants. (A) Assay by inverse PCR of *Rider* extrachromosomal circular DNA in drought-stressed wild-type plants and stressed *sit* mutants. Primer localization shown on the left (grey bar: *Rider* element, black box: LTR, arrowheads: PCR primers). Upper gel: specific PCR amplification of *Rider* circles after DNase treatment, lower gel: control PCR for *Rider* detection without DNase treatment. (B) Quantification of *Rider* DNA copy number, including both chromosomal and extrachromosomal copies, by qPCR in leaves of tomato plants subjected to drought-stress. Bar charts show normalized expression +/- SEM from two to three biological replicates. *P<0.05, two-sided Student’s *t*-test. (C) Assay by inverse PCR of *Rider* extrachromosomal circular DNA in *slnrpd1* and *slnrpe1* leaves. Upper gel: PCR amplification of *Rider* circles after DNase treatment, lower gel: control PCR for *Rider* detection without DNase treatment. (D) Quantification of *Rider* DNA copy number, including both chromosomal and extrachromosomal copies, by qPCR in *slnrpd1* and *slnrpe1* leaves. Bar charts show normalized expression +/- SEM from two to four biological replicates. *P<0.05, two-sided Student’s *t*-test. (E) Quantification of CHH DNA methylation levels at *Rider_08_3* and *Rider_07_2* in wild type, *slnrpd1* and *slnrpe1*. Levels expressed as % of methylated CHH sites. (F) Normalized siRNA count of 21-22-nt and 24-nt siRNAs at *Rider_08_3* and *Rider_07_2* in wild type, *slnrpd1* and *slnrpe1*. Data are expressed as siRNA reads per kb per million mapped reads.

The life cycle of LTR retrotransposons starts with transcription of the element, then the synthesis and maturation of accessory proteins including reverse transcriptase and integrase, reverse transcription, and the production of extrachromosomal linear (ecl) DNA that integrates into a new genomic location [61]. In addition, eclDNA can be a target of DNA repair and can be circularised by a non-homologous end-joining mechanism or homologous recombination between LTRs, resulting in extrachromosomal circular DNA (eccDNA) [62–65]. We searched for eccDNA to evaluate the consequences of increased *Rider* transcript accumulation due to drought stress or an impaired RdDM pathway on subsequent steps of the transposition cycle. After exonuclease-mediated elimination of linear dsDNA and circular ssDNA, *Rider* eccDNA was amplified by sequence-specific inverse PCR (Fig 4A). *Rider* eccDNA was absent in plants grown in control conditions but was detected in plants subjected to drought stress (Fig 4A). Sanger sequencing of the inverse PCR products showed that the amplified eccDNA probably originates from the *Rider_08_3* copy, which has 98.2% sequence homology of the 5’ and 3’ LTR sequences (S4A Fig). Residual sequence divergence may be due to genotypic differences between the reference genomic sequence and the genome of our experimental material. Analysis of CREs in the LTR of the eccDNA revealed the presence of all elements identified previously with the exception of a single nucleotide mutation located in the *CGCGBOXAT* box (S4A Fig).

Examination by quantitative PCR of the accumulation of *Rider* DNA, which included extrachromosomal and genomic copies, in drought-stressed plants also revealed an increase in *Rider* copy number due to eccDNA (Fig 4B). Importantly, *Rider* eccDNA was not detected in *sit* mutants subjected to drought stress (Fig 4A), suggesting that induced transcription of *Rider* by drought stress triggers production of extrachromosomal DNA and this response requires ABA biosynthesis.

We also examined the accumulation of *Rider* eccDNA in plants impaired in RdDM. Interestingly, *Rider* eccDNA was detected in *slnrpd1* and *slnrpe1* (Fig 4C) and increase in *Rider* DNA copy number due to eccDNA accumulation was confirmed by qPCR (Fig 4D). Absence of newly integrated genomic copies has been further validated by genome sequencing. The eccDNA forms differed between the mutants (Fig 4C). Sequencing of *Rider* eccDNA in *slnrpd1* showed a sequence identical to the *Rider* eccDNA of wild-type plants subjected to drought stress. Thus, the *Rider_08_3* copy is probably the main contributor to eccDNA in drought and in *slnrpd1*. In contrast, eccDNA recovered from *slnrpe1* had a shorter LTR (287 bp) and the highest sequence similarity with *Rider_07_2* (89.2%) (S4B Fig). Shortening of the LTR in this particular element results in the loss of the upstream *MYBCORE* as well as the *CGCGBOXAT* elements (S4B Fig).

We then asked whether DNA methylation and siRNA distribution at these particular *Rider* copies had changed in the mutants. DNA methylation at CHH sites, but not CG nor CHG, was drastically reduced at *Rider_08_3* in *slnrpd1* (Fig 4E, S4C–S4E Fig and S5A Fig) together with a complete loss of 24-nt siRNAs at this locus (Fig 4F and S4F Fig) but DNA methylation at *Rider_07_2* was not affected, despite the deficiency of SlNRPD1 or SlNRPE1 (Fig 4E, S4C–S4E Fig and S5B Fig). Levels of 21-22-nt siRNAs in both mutants and 24-nt siRNA in *slnrpe1* were increased (Fig 4F and S4F and S4G Fig). Altogether, this suggests that RdDM activity on *Rider* is highly copy-specific and that different components of the RdDM pathway differ in their effects on *Rider* silencing.

### *Rider* families in other plant species

**Fig 5:**
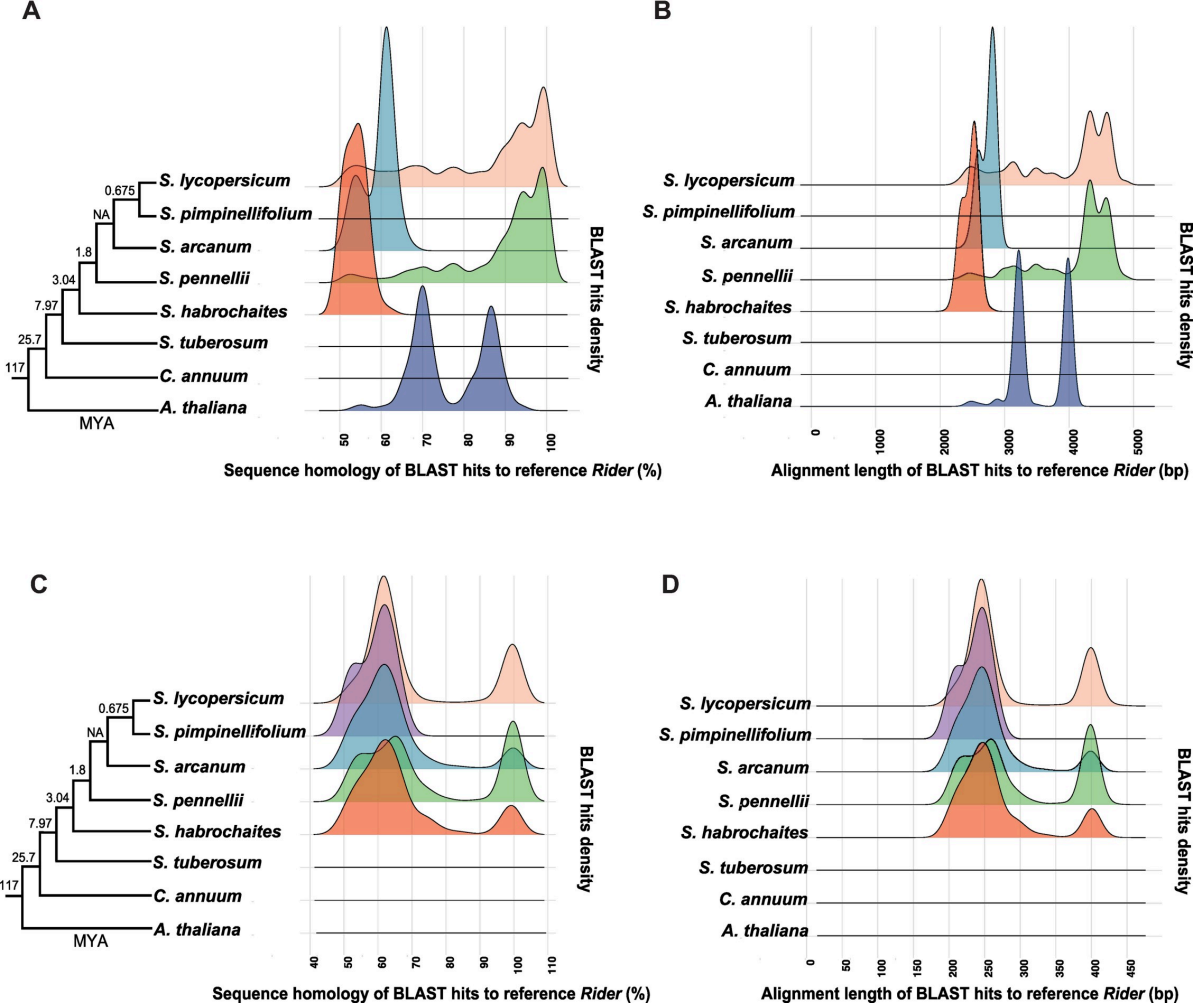
Distribution of *Rider* in other *Solanaceae* species. (A) *In silico* identification of *Rider* elements in *Solanaceae* species based on the density of high homology BLAST hits over the full-length reference *Rider* sequence. (B) Alignment length of high homology BLAST hits obtained in (A). (C) *In silico* identification of *Rider* elements in *Solanaceae* species based on the density of high homology BLAST hits over the reference *Rider* LTR sequence. (D) Alignment length of high homology BLAST hits obtained in (C). Left panels (A) and (C): phylogenetic trees of the species examined.

To examine the distribution of *Rider* retrotransposons in other plant species, we searched for *Rider*-related sequences across the genomes of further *Solanaceae* species, including wild tomatoes, potato (*Solanum tuberosum*), and pepper (*Capsicum annuum*). We used the *Rider* reference sequence [45] as the query against genome sequences of *Solanum arcanum*, *S*. *habrochaites*, *S*. *lycopersicum*, *S*. *pennellii*, *S*. *pimpinellifolium*, *S*. *tuberosum*, and *Capsicum annuum* (genome versions are listed in Materials and Methods). Two BLAST searches were performed, one using the entire *Rider* sequence as the query and the other using only the *Rider* LTR. Consistent with previous reports, *Rider-like* elements are present in wild relatives of tomato such as *S*. *arcanum*, *S*. *pennellii* and *S*. *habrochaites*; however, the homology levels and their lengths vary significantly between species (Fig 5A). While *S*. *arcanum* and *S*. *habrochaites* exhibit high peak densities at 55% and 61% homology, respectively, *S*. *pennellii* show a high peak density at 98% over the entire *Rider* reference sequence (Fig 5A). This suggests that the *S*. *arcanum* and *S*. *habrochaites* genomes harbour mostly *Rider-like* elements with relatively low sequence similarity, while *S*. *pennellii* retains full-length *Rider* elements.

To better visualize this situation, we aligned the BLAST hits to the reference *Rider* copy (Fig 5B). This confirmed that *Rider* elements in *S*. *pennellii* are indeed mostly full-length *Rider* homologs showing high density of hits throughout their lengths, while BLAST hits in the *S*. *arcanum* and *S*. *habrochaites* genomes showed only partial matches over the 4867 bp of the reference *Rider* sequence (Fig 5B). Unexpectedly, this approach failed to detect either full-length or truncated *Rider* homologs in the close relative of tomato, *S*. *pimpinellifolium*. Extension of the same approaches to the genomes of the evolutionary more distant *S*. *tuberosum* and *Capsicum annuum* failed to detect substantial *Rider* homologs (Fig 5A and 5B), confirming the absence of *Rider* in the potato and pepper genomes [45]. As a control, we also analysed *Arabidopsis thaliana*, since previous studies reported the presence of *Rider* homologs in this model plant [45]. Using the BLAST approach above, we repeated the results provided in [45] and found BLAST hits of high sequence homology to internal sequences of *Rider* in the *Arabidopsis thaliana* genome. However, we did not detect sequence homologies to *Rider* LTRs (Fig 5C and 5D). Motivated by this finding and the possibility that *Rider* homologs in other species may have highly divergent LTRs, we screened for *Rider* LTRs that would have been missed in the analysis shown in Fig 5A and 5B due to the use of the full-length sequence of *Rider* as the query. Using the *Rider* LTR as a query revealed that *S*. *pennellii*, *S*. *arcanum* and *S*. *habrochaites* retain intact *Rider* LTR homologs, but *S*. *pimpinellifolium* exhibits a high BLAST hit density exclusively at approximately 60% homology. This suggests strong divergence of *Rider* LTRs in this species (Fig 5C and 5D). Overall, the results indicate intact *Rider* homologs in some *Solanaceae* species, whereas sequence similarities to *Rider* occur only within the coding area of the retrotransposons in more distant plants such as *Arabidopsis thaliana*. Therefore, LTRs, which include the *cis*-regulatory elements conferring stress-responsiveness, diverge markedly between species. Finally, we performed a reciprocal BLAST against tomato using *Rider-like* hits from all other species having sequence similarity over the entire element between 50% - 84% and confirmed that all *Rider* loci in tomato were among the top reciprocal BLAST hits.

To address the specificity of this divergence in *Solanaceae* species, we examined whether the CREs enriched in *S*. *lycopersicum* (Fig 2A) are present in LTR sequences of the *Rider* elements in *S*. *pennellii*, *S*. *arcanum*, *S*. *habrochaites* and *S*. *pimpinellifolium* (Fig 5C). While the LTRs identified in *S*. *pennellii*, *S*. *arcanum* and *S*. *habrochaites* retained all five previously identified CREs, more distant LTRs showed shortening of the U3 region associated with loss of the CGCG box (S6 Fig and S5 Table). This was observed already in *S*. *pimpinellifolium*, where all identified *Rider* LTRs lacked part of the U3 region containing the CGCG box (S6 Fig). Thus, *Rider* distribution and associated features differ even between closely related *Solanaceae* species, correlated with the occurrence of a truncated U3 region and family-wide loss of CREs.

Finally, to test the evolutionary conservation of *Rider* elements across the plant kingdom, we performed *Rider* BLAST searches against all 110 plant genomes available at the NCBI Reference Sequence (RefSeq) database (www.ncbi.nlm.nih.gov/refseq). Using the entire *Rider* sequence as the query to measure the abundance of *Rider* homologs throughout these genomes, we found *Rider* homologs in 14 diverse plant species (S7 Fig). The limited conservation of *Rider* LTR sequences in the same 14 species, revealed using the LTR sequence as the query, suggests that *Rider* LTRs are highly polymorphic and that drought-responsive CREs may nevertheless be restricted to *Solanaceae* (S8 Fig).

## Discussion

### High-resolution map of full-length *Rider* elements in the tomato genome

Comprehensive analysis of individual LTR retrotransposon families in complex plant genomes has been facilitated and become more accurate with the increasing availability of high-quality genome assemblies. Here, we took advantage of the most recent tomato genome release (SL3.0) to characterize with improved resolution the high-copy-number *Rider* retrotransposon family. Although *Rider* activity has been causally linked to the emergence of important agronomic phenotypes in tomato, the triggers of *Rider* have remained elusive. Despite the relatively low proportion (approximately 20%) of euchromatic chromosomal regions in the tomato genome [32]), our *de novo* functional annotation of full-length *Rider* elements revealed preferential compartmentalization of recent *Rider* insertions within euchromatin compared to aged insertions. Mapping analyses further revealed that recent rather than aged *Rider* transposition events are more likely to modify the close vicinity of genes. However, *Rider* copies inserted into heterochromatin have been passively maintained for longer periods. This differs significantly from other retrotransposon families in tomato such as *Tnt1*, *ToRTL1* and *T135*, which show initial, preferential insertions into heterochromatic regions [66]. *TARE1*, a high-copy-number *Copia-like* element, is present predominantly in pericentromeric heterochromatin [67]. Another high-copy-number retrotransposon, *Jinling*, is also enriched in heterochromatic regions, making up about 2.5% of the tomato nuclear genome [68]. The *Rider* propensity to insert into gene-rich areas mirrors the insertional preferences of the *ONSEN* family in Arabidopsis. Since new *ONSEN* insertions confer heat-responsiveness to neighbouring genes [28,69], it is tempting to speculate that genes in the vicinity of new *Rider* insertions may acquire, at least transiently, drought-responsiveness.

### Environmental and epigenetic regulation of *Rider* activity

We found that *Rider* transcript levels are elevated during dehydration stress mediated by ABA-dependent signalling. The activation of retrotransposons upon environmental cues has been shown extensively to rely on the presence of *cis*-regulatory elements within the retrotransposon LTRs [48]. The heat-responsiveness of *ONSEN* in Arabidopsis [26,27,70], *Go-on* in rice [25], and *Copia* in Drosophila [71] is conferred by the presence in their LTRs of consensus sequences found in the promoters of heat-shock responsive genes. Thus, the host’s heat-stress signalling appears to induce transcriptional activation of the transposon and promote transposition [70]. While *ONSEN* and *Go-on* are transcriptionally inert in the absence of a triggering stress, transcripts of Drosophila *Copia* are found in control conditions, resembling the regulatory situation in *Rider*. Due to relatively high constitutive expression, increase in transcript levels of Drosophila *Copia* following stress appears modest compared to *ONSEN* or *Go-on*, which are virtually silent in control conditions [25–27,70]. Regulation of Drosophila *Copia* mirrors that of *Rider*, where transcript levels during dehydration stress are very high but the relative increase compared to control conditions is rather modest.

The presence of MYB recognition sequences within *Rider* LTRs suggests that MYB transcription factors participate in transcriptional activation of *Rider* during dehydration. Multiple MYB subfamilies are involved in ABA-dependent stress responses in tomato, but strong enrichment of the MYB core element CTGTTA within *Rider* LTRs suggests involvement of R2R3-MYB transcription factors, which are markedly amplified in *Solanaceae* [72]. Members of this MYB subfamily are involved in the ABA signalling-mediated drought-stress response [73] and salt-stress signalling [74]. This possible involvement of R2R3-MYBs in *Rider* is reminiscent of the transcriptional activation of the tobacco retrotransposon *Tto1* by the R2R3-MYB, member NtMYB2 [75]. Drought-responsiveness has been observed for *Rider_08_3* only, despite other individual *Rider* copies displaying intact MYB core element (S3 Table). This suggests that presence of this CRE is not the only feature required for drought-responsiveness, and other factors, such as genomic location, influence *Rider* activity. Indeed, *Rider_08_3* is located within a gene-rich area, with low TE content that might facilitate its activation. This is strikingly different from *Rider_07_2* that is nested in a TE-rich area and isolated from genes (S6 Table).

In addition to environmental triggers, *Rider* transcript levels are regulated by the RdDM pathway. Depletion of SlNRPD1 and SlNRPE1 increases *Rider* transcript abundance, resulting in production of extrachromosomal circular DNA. Analysis of *Rider*-specific siRNA populations revealed that siRNA targeting of *Rider* elements is mostly independent of their chromatin context. This is somewhat unexpected since RdDM activity in tomato seems to be restricted to gene-rich euchromatin and it was postulated that accessibility of RNA Pol IV to heterochromatin is hindered by the compact chromatin structure [60,76,77]. We identified *Rider* copies targeted by RdDM, which potentially influences local epigenetic features. Loss of SlNRPD1 and SlNRPE1 leads to over-accumulation of 21-22-nt siRNAs at *Rider* copies, suggesting that inactivation of canonical RdDM pathway-dependent transcriptional gene silencing triggers the activity of the non-canonical RDR6 RdDM pathway at *Rider* [78–80].

It is noteworthy that, despite clear effects on *Rider* transcript accumulation and siRNA accumulation, loss of SlNRPD1 and SlNRPE1 is not manifested by drastic changes in total DNA methylation levels of *Rider* at the family level. This is in accordance with the modest decrease in genome-wide CHH and CHG methylation described in tomato RdDM mutants, with most of the changes happening on the euchromatic arms while the pericentromeric heterochromatin is unaffected [60]. Distribution of the 71 intact *Rider* elements in both euchromatic and heterochromatic compartments thus likely hampers detection of major changes DNA methylation over the *Rider* family. Only young euchromatic *Rider* elements marginally lose CHH methylation in the *slnrpd1* mutant, but this is modest compared to the general decrease in mCHH observed throughout the chromosome arms [60]. As expected, CHH methylation at heterochromatic *Rider* is not affected. This suggests that SlCMT2 is involved in maintenance of mCHH at heterochromatic *Rider* copies in the absence of SlNRPD1, as observed previously for pericentromeric heterochromatin [60]. In general, our observations suggest that epigenetic silencing of *Rider* retrotransposons is particularly robust and involves compensatory pathways.

We identified extrachromosomal circular DNA originating from the *Rider* copies *Rider_08_3* and *Rider_07_2* in *slnrpd1* and *slnrpe1*, respectively. In terms of DNA methylation and siRNA distribution at these two specific copies, loss of SlNRPD1 and SlNRPE1 brought different copy-specific outcomes. *Rider_08_3*, the main contributor to eccDNA in *slnrpd1*, displayed a reduction in CHH methylation that may contribute to increased transcription and the accumulation of eccDNA. In *Rider_07_2*, that provides a template for eccDNA in *slnrpe1*, there was no change in DNA methylation levels. Therefore, transcription and the production of eccDNA from this *Rider* copy is not regulated by DNA methylation. Consequently, eccDNA from *Rider_07_2* was not detected in *slnrpd1* despite drastic loss of CHH methylation.

Despite our efforts, we were unable to apply either drought or ABA treatment to the *slnrpd1* and *slnrpe1* mutants. In contrast to Arabidopsis [81,82], RdDM mutants in tomato are showing severe developmental defects and are sterile [60]. They are particularly difficult to maintain even in optimal growth conditions, precluding the application of stress treatments. Altogether, it appears that transcriptional control and reverse transcription of *Rider* copies occurs via multiple layers of regulation, possibly specific for individual *Rider* elements according to age, sequence and genomic location, that are targeted by parallel silencing pathways, including non-canonical RdDM [83,84].

### *Rider* retrotransposons in other plant species

The presence of *Rider* in tomato relatives as well as in more distantly related plant species has been described previously [34,45,47]. However, the *de novo* identification of *Rider* elements in the sampling provided here shows the distribution of the *Rider* family within plant species to be more complex than initially suggested. Surprisingly, mining for sequences with high similarity, overlapping more than 85% of the entire reference sequence of *Rider*, detected no full-length *Rider* elements in *Solanum pimpinellifolium* but in all other wild tomato species tested. Furthermore, the significant accumulation of only partial *Rider* copies in *Solanum pimpinellifolium*, the closest relative of tomato, does not match the established phylogeny of the *Solanaceae*. The cause of these patterns is unresolved but two scenarios can be envisaged. First, the absence of full-length *Rider* elements may be due to the suboptimal quality of genome assembly that may exclude a significant proportion of highly repetitive sequences such as *Rider*. This is supported by the N50 values within the *Solanaceae*, where the quality of genome assemblies varies significantly between species, with *S*. *pimpinellifolium* showing the lowest (S7 Table). An improved genome assembly would allow a refined analysis of *Rider* in this species. Alternatively, active *Rider* copies may have been lost in *S*. *pimpinellifolium* since the separation from the last common ancestor but not in the *S*. *lycopersicum* and *S*. *pennellii* lineages. The high-density of solo-LTRs and truncated elements in *S*. *pimpinellifolium* is in agreement with this hypothesis.

Comparing the sequences of *Rider* LTRs in the five tomato species, the unique occurrence of LTRs lacking most of the U3 region in *S*. *pimpinellifolium* suggests that loss of important regulatory sequences has impeded maintenance of intact *Rider* elements. Interestingly, part of the U3 region missing in *S*. *pimpinellifolium* contains the CGCG box, which is involved in response to environmental signals [49], as well as a short CpG-island-like structure (position 52–155 bp on reference *Rider*). CpG islands are usually enriched 5’ of transcriptionally active genes in vertebrates [85] and plants [86]. Despite the presence of truncated *Rider* LTRs, the occurrence of intact, full-length LTRs in other wild tomato species indicates that *Rider* is still potentially active in these genomes.

Altogether, our findings suggest that inter- and intra-species TE distribution can be uncoupled and that the evolution of TE families in present crop plants was more complex than initially anticipated. We have further opened interesting perspectives for harnessing transposon activities in crop breeding. Potentially active TE families that react to environmental stimuli, such as *Rider*, provide an unprecedented opportunity to generate genetic and epigenetic variation from which desirable agronomical traits may emerge. Notably, rewiring of gene expression networks regulating the drought-stress responses of new *Rider* insertions is an interesting strategy to engineer drought-resilient crops.

## Materials and methods

### Plant material and growth conditions

Tomato plants were grown under standard greenhouse conditions (16 h at 25°C with supplemental lighting of 88 w/m^2^ and 8 h at 15°C without). *flacca* (*flc*), *notabilis* (*not*), and *sitiens* (*sit*) seeds (cv. Ailsa Craig) were obtained from Andrew Thompson, Cranfield University; the *slnrpd1* and *slnrpe1* plants (cv. M82) were described before [60]. For aseptic growth, seeds of *Solanum lycopersicum* were surface-sterilized in 20% bleach for 10 min, rinsed three times with sterile H_2_O, germinated and grown on half-strength MS media (16 h light and 8 h dark at 24°C).

### Stress treatments

For dehydration stress, two-week-old greenhouse-grown plants were subjected to water deprivation for two weeks. For NaCl and mannitol treatments, tomato seedlings were grown aseptically for two weeks prior to transfer into half-strength MS solution containing 100, 200 or 300 nM NaCl or mannitol (Sigma) for 24 h. For abscisic acid (ABA) treatments, tomato seedlings were grown aseptically for two weeks prior to transfer into half-strength MS solution containing 0.5, 5, 10 or 100 μM ABA (Sigma) for 24 h. For 5-azacytidine treatments, tomato seedlings were germinated and grown aseptically on half-strength MS media containing 50 nM 5-azacytidine (Sigma) for two weeks. For cold stress experiments, two-week-old aseptically grown plants were transferred to 4°C for 24 h prior to sampling.

### RNA extraction and quantitative RT-PCR analysis

Total RNA was extracted from 200 mg quick-frozen tissue using the TRI Reagent (Sigma) according to the manufacturer’s instructions and resuspended in 50 μL H_2_O. The RNA concentration was estimated using the Qubit Fluorometric Quantitation system (Thermo Fisher). cDNAs were synthesized using a SuperScript VILO cDNA Synthesis Kit (Invitrogen). Real-time quantitative PCR was performed in the LightCycler 480 system (Roche) using primers listed in S8 Table. Selected *Rider* primers amplify 64 out of the 71 copies, with 3 mismatches allowed. Localization of *Rider* primers is shown in S9 Fig. LightCycler 480 SYBR Green I Master premix (Roche) was used to prepare the reaction mixture in a volume of 10 μL. Transcript levels were normalized to *SlACTIN* (*Solyc03g078400*). The results were analysed by the ΔΔCt method.

### DNA extraction and copy number quantification

Tomato DNA was extracted using the Qiagen DNeasy Plant Mini Kit (Qiagen) following the manufacturer’s instructions and resuspended in 30 μL H_2_O. DNA concentration was estimated using the Qubit Fluorometric Quantitation system (Thermo Fisher). Quantitative PCR was performed in the LightCycler 480 system (Roche) using primers listed in S8 Table. Selected *Rider* primers amplify 64 out of the 71 copies, with 3 mismatches allowed. Localization of *Rider* primers is shown in S9 Fig. LightCycler 480 SYBR Green I Master premix (Roche) was used to prepare the reaction in a volume of 10 μL. DNA copy number was normalized to *SlACTIN* (*Solyc03g078400*). Results were analysed by the ΔΔCt method.

### Extrachromosomal circular DNA detection

Extrachromosomal circular DNA amplification was derived from the previously published mobilome analysis [11]. In brief, extrachromosomal circular DNA was separated from chromosomal DNA using PlasmidSafe ATP-dependent DNase (EpiCentre) according to the manufacturer’s instructions with the incubation at 37°C extended to 17 h. The PlasmidSafe exonuclease degrades linear DNA and thus safeguards circular DNA molecules. Circular DNA was precipitated overnight at -20°C in 0.1 v/v 3 M sodium acetate (pH 5.2), 2.5 v/v EtOH and 1 μL glycogen (Sigma). The pellet was resuspended in 20 μL H_2_O. Inverse PCR reactions were carried out with 2 μL of DNA solution in a final volume of 20 μL using the GoTaq enzyme (Promega). The PCR conditions were as follows: denaturation at 95°C for 5 min, followed by 30 cycles at 95°C for 30 s, an annealing step for 30 s, an elongation step at 72°C for 60 s, and a final extension step at 72°C for 5 min. Selected primers amplify 68 out of the 71 *Rider* copies, with 3 mismatches allowed. Primer localization is shown on Fig 4A and 4C, left panel (grey bar: *Rider* element, black box: LTR, arrowheads: PCR primers) and sequences are listed in S8 Table. PCR products were separated in 1% agarose gels and developed by NuGenius (Syngene). Bands were extracted using the Qiagen Gel Extraction Kit and eluted in 30 μL H_2_O. Purified amplicons were subjected to Sanger sequencing. Five amplicons, obtained from two independent experiments, were sequenced for each eccDNA form.

### Phylogenetic analysis of de novo identified *Rider* elements

A phylogenetic tree was constructed from the nucleotide sequences of the 71 *Rider* elements using Geneious 9.1.8 (www.geneious.com) and built with the Tamura-Nei neighbor joining method. Pairwise alignment for the building distance matrix was obtained using a global alignment with free end gaps and a cost matrix of 51% similarity.

### Distribution analysis

Genomic coordinates of each of the 71 *Rider* elements identified by *de novo* annotation using *LTRpred* (https://github.com/HajkD/LTRpred) have been used to establish their chromosomal locations. Coordinates for centromeres were provided before [32] and pericentromeric regions were defined by high levels of DNA methylation and H3K9me2 ([60] and David Baulcombe, personal communication).

### Accession numbers

The Genbank accession number of the reference *Rider* nucleotide sequence identified in [45] is EU195798.2. We used *Solanum lycopersicum* bisulfite and small RNA sequencing data (SRP081115) generated in [60].

### Dating of insertion time

Insertion times of *Rider* elements were estimated using the method described in [45]. Degrees of divergence between LTRs of each individual element were determined using *LTRpred*. LTR divergence rates were then converted into dates using the average substitution rate of 6.96 x 10^−9^ substitutions per synonymous site per year for tomato [87].

### Bisulfite sequencing analysis

We collected data from previously published BS-seq libraries of tomato mutants of RNA polymerase IV and V and controls [60]: *slnrpe1* (SRR4013319), *slnrpd1* (SRR4013316), wild type *CAS9* (SRR4013314) and not transformed wild type (SRR4013312). The raw reads were analysed using our previously established pipeline [88] and aligned to the *Solanum lycopersicum* reference version SL3.0 (www.solgenomics.net/organism/Solanum_lycopersicum/genome). The chloroplast sequence (NC_007898) was used to estimate the bisulfite conversion (on average above 99%). The R package DMRcaller [89] was used to summarize the level of DNA methylation in the three cytosine contexts for each *Rider* copy.

### Small RNA sequencing analysis

Tomato siRNA libraries were obtained from [60] and analysed using the same analysis pipeline to align reads to the tomato genome version SL3.0. Briefly, the reads were trimmed with Trim Galore! (www.bioinformatics.babraham.ac.uk/projects/trim_galore) and mapped using the ShortStack software v3.6 [90]. The siRNA counts on the loci overlapping *Rider* copies were calculated with R and the package GenomicRanges.

### Genome sequence data

Computationally reproducible analysis and annotation scripts for the following sections can be found at http://github.com/HajkD/RIDER.

### Genomic data retrieval

We retrieved genome assemblies for 110 plant species (S9 Table) from NCBI RefSeq [91] using the *meta*.*retrieval* function from the R package *biomartr* [92]. For *Solanum lycopersicum*, we retrieved the most recent genome assembly version SL3.0 from the *Sol Genomics Network ftp*:*//ftp*.*solgenomics*.*net/tomato_genome/assembly/build_3*.*00/S_lycopersicum_chromosomes*.*3*.*00*.*fa* [93].

### Functional *de novo* annotation of LTR retrotransposons in *Solanaceae* genomes

Functional *de novo* annotations of LTR retrotransposons for seventeen genomes from the *Asterids*, *Rosids*, and *monocot* clades (*Asterids*: *Capsicum annuum*, *C*. *baccatum MLFT02_5*, *C*. *chinense MCIT02_5*, *Coffea canephora*, *Petunia axillaris*, *Phytophthora inflata*, *Solanum arcanum*, *S*. *habrochaites*, *S*. *lycopersicum*, *S*. *melongena*, *S*. *pennellii*, *S*. *pimpinellifolium*, *S*. *tuberosum*; *Rosids*: *Arabidopsis thaliana*, *Vitis vinifera*, and *Cucumis melo*; *Monocots*: *Oryza sativa*) were generated using the *LTRpred*.*meta* function from the *LTRpred* annotation pipeline (https://github.com/HajkD/LTRpred; also used in [25]). To retrieve a consistent and comparable set of functional annotations for all genomes, we consistently applied the following *LTRpred* parameter configurations to all *Solanaceae* genomes: minlenltr = 100, maxlenltr = 5000, mindistltr = 4000, maxdisltr = 30000, mintsd = 3, maxtsd = 20, vic = 80, overlaps = “no”, xdrop = 7, motifmis = 1, pbsradius = 60, pbsalilen = c(8,40), pbsoffset = c(0,10), quality.filter = TRUE, n.orf = 0. The plant-specific tRNAs used to screen for primer binding sites (PBS) were retrieved from GtRNAdb [94] and plant RNA [95] and combined in a custom *fasta* file. The hidden Markov model files for gag and pol protein conservation screening were retrieved from Pfam [96] using the protein domains RdRP_1 (PF00680), RdRP_2 (PF00978), RdRP_3 (PF00998), RdRP_4 (PF02123), RVT_1 (PF00078), RVT_2 (PF07727), Integrase DNA binding domain (PF00552), Integrase zinc binding domain (PF02022), Retrotrans_gag (PF03732), RNase H (PF00075), and Integrase core domain (PF00665).

### Sequence clustering of functional LTR retrotransposons from 17 genomes

We combined the *de novo* annotated LTR retrotransposons of the 17 species mentioned in the previous section in a large fasta file and used the cluster program *VSEARCH* [97] with parameter configurations: *vsearch—cluster_fast—qmask none–id 0*.*85—clusterout_sort—clusterout_id—strand both—blast6out—sizeout* to cluster LTR retrotransposons by nucleotide sequence homology (global sequence alignments). Next, we retrieved the 85% sequence homology clusters from the *VSEARCH* output and screened for clusters containing *Rider* sequences. This procedure enabled us to detect high sequence homology (>85%) sequences of *Rider* across diverse species.

### Nucleotide BLAST search of *Rider* against 110 plant genomes

To determine the distribution of *Rider* related sequences across the plant kingdom, we performed BLASTN [98] searches of *Rider* (= query sequence) using the function *blast_genomes* from the R package *metablastr* (https://github.com/HajkD/metablastr) against 110 plant genomes (S9 Table) and the parameter configuration: *blastn -eval 1E-5 -max_target_seqs 5000*. As a result, we retrieved a BLAST hit table containing 11,748,202 BLAST hits. Next, we filtered for hits that contained at least 50% sequence coverage (= sequence homology) and throughout at least 50% sequence length homology to the reference *Rider* sequence. This procedure reduced the initial 11,748,202 BLAST hits to 57,845 hits, which we further refer to as *Rider-like* elements. These 57,845 *Rider-like* elements are distributed across 21 species with various abundance frequencies. In a second step, we performed an analogous BLASTN search using only the 5’ LTR sequence of *Rider* to determine the distribution of *Rider-like* LTR across the plant kingdom. Using the same BLASTN search strategy described above, we retrieved 9,431 hits. After filtering for hits that contained at least 50% percent sequence coverage (= sequence homology) and at least 50% sequence length homology to the reference *Rider* LTR sequence, we obtained 2,342 BLAST hits distributed across five species.

### Motif enrichment analysis

We tested the enrichment of *cis*-regulatory elements (CREs) in *Rider* using two approaches. In the first approach, we compared *Rider* CREs to promoter sequences of all 35,092 protein coding genes from the tomato reference genome. We retrieved promoter sequences 400 bp upstream of the TSS of the respective genes. We constructed a 2x2 contingency table containing the respective motif count data of CRE observations in true *Rider* sequences *versus* counts in promoter sequences. We performed a Fisher’s exact test for count data to assess the statistical significance of enrichment between the motif count data retrieved from *Rider* sequences and the motif count data retrieved from promoter sequences. In the second approach, due to the unavailability of gene annotation for *Solanum arcanum*, *Solanum habrochaites* and *Solanum pimpinellifolium* we compared *Rider* CREs to randomly sampled sequence loci from the same genome using the following two step procedure: in step one, we sampled 1000 DNA sequences with the same length as the reference *Rider* sequence from 1000 randomly sampled loci in the tomato reference genome. When sampling, we also considered the strand direction of the reference *Rider* sequence. Whenever a *Rider* sequence was annotated in the plus direction, we also sampled the corresponding set of random sequences in the plus direction of the respective randomly drawn locus. In contrast, when a *Rider* sequence was annotated in the minus direction, we also sampled the corresponding set of random sequences in the minus direction. In step two, we counted CRE occurrences for each *Rider* sequence independently and for a set of different CREs. Next, we counted the number of the same CRE occurrences for each random sequence independently to assess how often these CREs were found in random sequences. We then, analogous to the first approach, constructed a 2x2 contingency table containing the respective motif count data of CRE observations in true *Rider* sequences *versus* counts in random sequences. We performed a Fisher’s exact test for count data to assess the statistical significance of enrichment between the motif count data retrieved from *Rider* sequences and the motif count data retrieved from random sequences. The resulting *P*-values are shown in S4 Table for the first approach and in S5 Table for the second approach. Computationally reproducible scripts to perform the motif count analysis can be found at https://github.com/HajkD/RIDER.

### Calculation of N50 metric

To assess the genome quality of *Solanaceae* species, we calculated the N50 metric for the genome assemblies of *Solanum lycopersicum*, *S*. *pimpinellifolium*, *S*. *arcanum*, *S*. *pennellii*, *S*. *habrochaites*, and *S*. *tuberosum* using the following procedure. First, we imported the scaffolds or chromosomes of each respective genome assembly using the R function *read_genome()* from the *biomartr* package. Next, for each species individually we determined the sequence length for each scaffold or chromosome and sorted them according to length in descending order. The N50 value in Mbp was then calculated in R as follows: N50 <- len.sorted[cumsum(len.sorted) > = sum(len.sorted)*0.5][1] / 1000000, where the variable *len*.*sorted* denotes the vector storing the ordered scaffold or chromosome lengths of a genome assembly.

### Availability of data and materials

SRAtoolkit, v2.8.0 (https://github.com/ncbi/sra-tools) and Biomartr 0.9.9000 (https://ropensci.github.io/biomartr/index.html) were used for data collection.

Phylogenetic trees were constructed using Geneious 9.1.8 (www.geneious.com).

The *de novo* retrotransposon annotation pipeline *LTRpred* is available in the GitHub repository (https://github.com/HajkD/LTRpred).

*Rider* annotation and analysis pipeline is available in the GitHub repository (https://github.com/HajkD/RIDER).

Distribution of *Rider* elements was done using the R package *metablastr* (https://github.com/HajkD/metablastr).

DNA methylation levels were assessed using the R package DMRcaller (http://bioconductor.org/packages/release/bioc/html/DMRcaller.html).

Small RNA analysis was done using Trim Galore! (www.bioinformatics.babraham.ac.uk/projects/trim_galore), ShortStack v3.6 (https://github.com/MikeAxtell/ShortStack) and GenomicRanges v3.8 (https://bioconductor.org/packages/release/bioc/html/GenomicRanges.html).

Reference *Rider* nucleotide sequence (accession number EU195798) is available here (https://www.ncbi.nlm.nih.gov/nuccore/EU195798).

The datasets supporting the conclusions of this article are available at Sequence Read Archive (SRA) (https://www.ncbi.nlm.nih.gov/sra/) under accession numbers "SRP081115", "SRR4013319", "SRR4013316", "SRR4013314" and "SRR4013312".

## Supporting information

S1 Fig Distribution of 71 *de novo* annotated *Rider* elements based on LTR similarity and chromatin context. (A) Age distribution of total *Rider* elements based on LTR similarity and corresponding classes. (B) Age distribution of *Rider* elements inserted in heterochromatic (HC) and euchromatic (EC) regions based on LTR similarity.
(EPS)

S2 Fig *Rider* transcripts levels are unaffected by cold stress. (A-D) Quantification of *SlASR1* RNA levels by RT-qPCR in wild-type tomato seedlings after (A) drought stress (B) mannitol, (C) NaCl or (D) ABA treatments. (E) Quantification of *SlASR1* RNA levels in leaves of drought-stressed tomato wild-type plants, *flc*, *not* and *sit* mutants. (F) Quantification of *SlASR1* RNA levels by RT-qPCR in wild-type tomato seedlings after 5-azacytidine and ABA treatments. (G) Quantification of *Rider* RNA levels by RT-qPCR in wild-type tomato seedlings after cold stress. Bar charts show normalized expression +/- SEM from three to five biological replicates.
(EPS)

S3 Fig Distribution of siRNAs and DNA methylation within *Rider* sub-groups. (A) 21-22-nt and (B) 24-nt siRNAs normalized counts at distinct *Rider* sub-groups in wild type, wild type with *CAS9*, *slnrpd1* and *slnrpe1*. *Rider* elements are classified based on LTR similarity (80–95%, 95–98% and 98–100%), while *Rider* (Euchromatin) denotes copies located on euchromatic arms and *Rider* (Heterochromatin) copies located in pericentromeric heterochromatin. Data are expressed as siRNA reads per kb per million mapped reads, and represent average normalized siRNA counts on *Rider* elements +/- SD from *Rider* copies in the sub-group. (C) Quantification of DNA methylation levels in the CG, CHG and CHH contexts at *Rider* in wild type, *slnrpd1* and *slnrpe1*. The levels are averages of DNA methylation (%) in each context over the 71 *de novo* annotated *Rider* copies. (D) Quantification of CHH DNA methylation levels at *Rider* sub-groups in wild type, *slnrpd1* and *slnrpe1*. The levels are averages of DNA methylation (%) in the CHH context over *Rider* sub-groups.
(EPS)

S4 Fig Distinct *Rider* copies contribute to the production of extrachromosomal circular DNA. Comparison of the LTR nucleotide sequence from *Rider* extrachromosomal circular DNA detected after drought, or in *slnrpd1* (A) or *slnrpe1* (B), with the reference *Rider* LTR using EMBOSS Needle (www.ebi.ac.uk/Tools/psa/emboss_needle). CREs are marked as coloured boxes. (C) Quantification of CHH DNA methylation levels at LTRs and body of *Rider_08_3* and *Rider_07_2* in wild type, *slnrpd1* and *slnrpe1*. Levels expressed as % of methylated CHH sites. (D-E) Quantification of CG (D) and CHG (E) contexts DNA methylation levels at *Rider_08_3* and *Rider_07_2* in wild type, *slnrpd1* and *slnrpe1*. Levels expressed as % of methylated sites. (F-G) Normalized siRNA count of 24-nt (F) and 21-22-nt (G) siRNAs at LTRs and body of *Rider_08_3* and *Rider_07_2* in wild type, *slnrpd1* and *slnrpe1*. Data are expressed as siRNA reads per kb per million mapped reads.
(EPS)

S5 Fig DNA methylation status of *Rider* elements producing eccDNA. DNA methylation status of *Rider_08_3* (A) and *Rider_07_2* (B). Snapshots from IGV are shown. CG (blue track), CHG (red track) and CHH (green track) methylation coverage are shown for WT, *slnrpd1* and *slnrpe1*. *Rider* is identified as a blue block in the *LTRpred* track.
(EPS)

S6 Fig Characterization of *Rider* sub-populations in *Solanaceae* based on LTR sequences. Coverage over reference *Rider* LTR of high homology sequences identified by BLAST in Fig 5C. Sequences classified as “long LTR” were selected by filtering for BLAST hits with alignment lengths between 350–450 bp and >50% sequence and length homology to reference *Rider*. Sequences classified as “short LTR” were selected by filtering for BLAST hits with alignment lengths between 150–300 bp and >50% sequence and length homology to reference *Rider*.
(EPS)

S7 Fig Identification of *Rider* homologs in 14 plant species. *In silico* identification of *Rider* homologs in 14 plant species based on the density of high homology BLAST hits over the full-length reference *Rider* sequence (left) and alignment length of BLAST hits obtained (right). Species are ordered by evolutionary distance to *Solanum lycopersicum* according to www.timetree.org, www.genome.jp and references in S1 Text.
(EPS)

S8 Fig Non-*Solanaceae Rider* homologs lack LTR sequence conservation. *In silico* identification of *Rider* LTR homologs in 14 plant species based on the density of high homology BLAST hits over the reference *Rider* LTR sequence only. Species are ordered by evolutionary distance to *Solanum lycopersicum* according to www.timetree.org, www.genome.jp and references in S1 Text.
(EPS)

S9 Fig Localization of primers within *Rider* LTRs. Binding sites of qPCR primers within *Rider* LTRs are shown as purple arrows. *Rider* LTR U3, R and U5 regions are marked, as well as neighbouring Target Site Duplication (TSD) and Primer Binding Site (PBS) sequences. CREs are marked as coloured vertical bars.
(EPS)

S1 Table *De novo* annotation of LTR retrotransposons in the SL3.0 genome by *LTRpred*. (XLSX)

S2 Table Patristic distances between 71 *de novo* annotated *Rider* copies. (XLSX)

S3 Table Presence of *cis*-regulatory elements in individual *Rider* copies. (XLSX)

S4 Table Identification and enrichment analysis of *cis*-regulatory elements in *Rider* LTRs. (XLSX)

S5 Table Enrichment analysis of *cis*-regulatory elements in *Rider* LTRs in four *Solanaceae* species. (XLSX)

S6 Table Characteristics of *Rider_08_3* and *Rider_07_2* loci. (XLSX)

S7 Table N50 metric for six *Solanaceae* species. (XLSX)

S8 Table Primers used in this study. (XLSX)

S9 Table List of the 110 plant genomes used for the large-scale *Rider* BLAST search. (XLSX)

S1 Text Supporting references. (DOCX)

S1 Data Supporting numerical data. (XLSX)
