# Supplementary material for: Environmental and epigenetic regulation of Rider retrotransposons in tomato
Source: PLoS Genet. 2019 Sep 16;15(9):e1008370. doi: 10.1371/journal.pgen.1008370 (PMC6762207; doi:10.1371/journal.pgen.1008370)
Supplement: S1 Text — (DOCX) [file pgen.1008370.s019.docx]

**S1 Text: Supporting references**

Harkess, A. *et al.* The asparagus genome sheds light on the origin and evolution of a young y chromosome. *Nat. Commun.* **8,** (2017).

Zou, C. *et al.* A high-quality genome assembly of quinoa provides insights into the molecular basis of salt bladder-based salinity tolerance and the exceptional nutritional value. *Cell Res.* **27,** 1327–1340 (2017).
